# Evaluating the relationship between caregiver depression, social support, and children’s internalizing and externalizing symptoms in families affected by 22q11.2 deletion syndrome

**DOI:** 10.1186/s13023-025-03955-2

**Published:** 2025-08-18

**Authors:** Holly Carbyn, Abiaz Hossain, Raquel L. Dias, Lisa D. Palmer, Sophie Ayoub, Patricia Lingley-Pottie, Patrick J. McGrath, Andrea L. Rideout, Andrea Shugar, Cheryl Cytrynbaum, Donna M. McDonald-McGinn, Ann Swillen, Anne S. Bassett, Sandra Meier

**Affiliations:** 1 Department of Psychiatry, Dalhousie University, Halifax, NS Canada; 2 The Dalgish Family 22Q Clinic, Department of Psychiatry and Division of Cardiology, Department of Medicine, and Toronto General Hospital Research Institute, University Health Network, Toronto, ON Canada; 3 Institute for Biomedical Ethics, University of Basel, Basel, Switzerland; 4 IWK Health Centre Department of Psychiatry and Specific Care Clinics, 5850/5980 University Ave., PO Box 9700, Halifax, NS B3K 6R8 Canada; 5 Division of Clinical & Metabolic Genetics and the Department of Genetic Counselling, The Hospital for Sick Children, Toronto, ON Canada; 6 Division of Human Genetics, 22Q and You Center, Clinical Genetics Center, and Section of Genetic Counselling, Children’s Hospital of Philadelphia, Philadelphia, USA; 7 Department of Pediatrics, Perelman School of Medicine, University of Pennsylvania, Philadelphia, PA USA; 8 Center for Human Genetics, UZ Leuven, Leuven, Belgium; 9 Department of Human Genetics, Leuven, KU Belgium

**Keywords:** 22q11.2 deletion syndrome, Mental health, Depression, Social support, Mediation

## Abstract

**Background:**

22q11.2 Deletion Syndrome (22q11DS) is the most common microdeletion syndrome. It exhibits broad phenotypic variability, often including conditions like autism spectrum disorder and intellectual disability. Caregivers of children with 22q11DS are known to be at increased risk of poor mental health and less social support, which might affect their children’s health. The current study examined the relationship between parental mental health, perceived social support, and child mental health (internalizing/externalizing symptoms) in children with 22q11DS.

**Method:**

Ninety caregivers of children with 22q11DS completed an online survey measuring parental depressive symptoms, perceived social support, and child mental health problems (internalizing and externalizing symptoms). Structural equation models were run to examine the postulated relationships between variables.

**Results:**

The caregiver’s depressive symptoms were associated with higher internalizing and externalizing symptoms in their children with 22q11DS (mean age 11.8, SD 7.6 years). Caregivers experiencing symptoms of depression were less likely to report strong social support, and lower perceived social support was associated with greater child internalizing and externalizing symptoms. The relationship between caregiver’s depressive symptoms and internalizing symptoms in their children was mediated by perceived social support, but no such mediating effects were observed for externalizing symptoms.

**Conclusions:**

These findings provide valuable insights into the mental health burdens facing families living with 22q11DS. Interventions focusing on 22q11DS should integrate techniques to foster social and other supports to improve the mental health of caregivers and children.

## Introduction

The 22q11.2 deletion syndrome (22q11DS) is the most common microdeletion syndrome, present in about 1 in 2000 live births [1–3]. It is caused by a spontaneous hemizygous deletion on the long arm of chromosome 22 [4], affecting nearly every bodily system, including the central nervous system [1–3]. This results in a highly variable phenotype, ranging from life-threatening conditions to mild/atypical features, often delaying genetic diagnosis [6]. Developmental delays and learning difficulties affect 70–90% of individuals with 22q11DS. They also experience high rates of psychiatric illnesses; 25% of adults with 22q11DS develop schizophrenia, and many individuals have anxiety disorders, attention deficit hyperactivity disorder (ADHD), autism spectrum disorder (ASD), perseveration, and social difficulties [1–4, 7, 8]. Developmental health challenges such as intellectual disabilities, learning difficulties, ASD, and ADHD are often associated with increased mental health and behavioural and emotional challenges [7, 9]. In children, these symptoms may be characterized by two broad groups: Internalizing symptoms (worry, anxiety, depression, social withdrawal) and externalizing symptoms (hyperactivity, impulsivity, aggression, defiance) [10]. Youth with 22q11DS show significantly higher levels of internalizing and externalizing symptoms than the general population, with increased symptoms over time [11]. Internalizing symptoms are more common than externalizing symptoms, especially at older ages [8, 12, 13]. In individuals with 22q11DS, high levels of internalizing and externalizing symptoms may represent prodromal symptoms of treatable psychotic illness [12, 14], suggesting the importance of identifying possible mitigation strategies.

Beyond individual challenges, 22q11DS can impact the patient’s caregivers, who report increased stress, struggles to manage their child’s behaviour, strain on familial relationships, and financial difficulties [15, 16]. Specifically, mothers of children with 22q11DS are at an increased risk of depression [17, 18] compared to the general adult population [19]. Depression impacts everyday functioning, often exacerbating family conflicts, and affecting communication, emotional responses, and problem-solving abilities [27]. Importantly, social support has been shown to reduce depressive symptoms likely by enhancing self-worth, mood, and resilience to stress through positive interactions, assistance, and a sense of belonging [20–23]. Among parents of children with disabilities, strong social support is linked to lower depression levels, even under high stress [24, 25]. However, caregivers of children with 22q11DS often feel isolated due to limited social support, a challenge worsened by institutional and community stigma, leaving families feeling judged and unsupported [16, 26]. In 22q11DS, caregiver depression significantly affects child’s mental health, with higher parental anxiety and depression linked to increased internalizing and externalizing symptoms [28]. Children often develop cognitive, behavioural, and social skills by observing and mimicking their parents [29, 30]. Parents with depressive symptoms may struggle to seek social support, modeling ineffective coping strategies their children may adopt, while those who demonstrate healthy coping with strong social support foster effective coping strategies in their children [29–32] This lack of modelled support-seeking can leave children with unmet social support needs, increasing their risk for both internalizing and externalizing symptoms [25].

Parental social support can sometimes explain the relationship between parental depressive symptoms and child internalizing/externalizing symptoms. Koverola et al. found that maternal social support mediated the relationship between maternal victimization (highly correlated with depression) and child externalizing symptoms [33]. In 2006, Lee et al. reported that high social support buffered the negative effects of maternal depression on child externalizing symptoms, while childcare (arguably a form of social support) lessened its effect on internalizing symptoms [34]. Similarly, in a longitudinal study, maternal social support acted as a mediator between parental depressive symptoms and children’s internalizing symptoms over seven years [25]. Given research for other populations, we predict that greater levels of social support for parents and better caregiver mental health may be associated with lower levels of internalizing and externalizing symptoms in children with 22q11DS. In particular, the potential buffering effect of social support on the relationship between caregiver and offspring mental health in 22q11DS has not yet been studied.

## Objectives

To examine the mediating effect of social support on the mental health of families affected by 22q11DS, we first tested whether caregivers’ depressive symptoms were associated with internalizing and externalizing symptoms in children with 22q11DS. Next, we focused on our main objective and evaluated whether the relationship between caregivers’ depressive symptoms and child internalizing/externalizing symptoms was mediated by caregivers’ perceived social support levels. In line with previous findings in 22q11DS [28], we expected that higher levels of depressive symptoms in caregivers would be linked to increased internalizing and externalizing symptoms in their children with 22q11DS. Based on the observed buffering effect of social support in other populations [25, 33, 34], we expected that caregiver-reported social support would similarly mediate the association between caregiver depressive symptoms and internalizing/externalizing symptomatology in the child with 22q11DS.

## Methods

### Study design and data collection

The data for this study were gathered via a cross-sectional survey conducted between December 2021 and June 2022 for caregivers of a family member with 22q11DS. Participants were recruited through advertisements containing direct links to the study, distributed using social media platforms (Instagram, Facebook, and Twitter) to reach online support communities. Additionally, advertisements were posted, and emails were used to spread information to potential participants by the IWK Health Centre (Halifax, Nova Scotia) and the Hospital for Sick Children’s (SickKids) 22q11DS clinic (Toronto, Ontario).

Eligible caregiver participants were 1) over 18 years of age, 2) providing care for a family member with 22q11DS, 3) living in Canada/United States/Europe, and 4) able to read English and provide written consent. Eligibility was determined by self-report, with no exclusion criteria (including parental genetic status, and these data were not collected).

Caregivers first completed a screening questionnaire online. Eligible participants were next directed to a detailed self-guided consent process. Then the participants were asked to complete an online survey including: demographic information, mental health questionnaires, a parenting questionnaire and questions about their child’s behaviour. The screening, consent and survey were hosted on Research Electronic Data Capture (REDCap) recruitment site housed by Dalhousie University. REDCap is a free and secure application for data collection approved by Canadian and European healthcare authorities [35].

### Measures

#### Demographics

We collected 16 items pertaining to the caregiver’s characteristics and their family member with 22q11DS (see Table 1). The questionnaire was developed amongst clinicians and caregivers to ensure questions were clear, comprehensive and captured relevant information [36].

**Table 1:** Socio-demographic characteristics of the sample (N = 90)

Characteristics	N	%
*Caregiver’s gender*		
Woman	88	97.8
Man	2	2.2
*Caregiver’s age*		
Range	29–73	
Mean(SD)	49.3 (9.3)	
*Country*		
USA	51	56.7
Canada	35	38.9
Europe	4	4.4
*Child’s age (in years)*		
0–12	56	62.2
13–17	21	23.3
18 +	13	14.4
Range	1–42	
Mean(SD)	11.8 (7.6)	
Median	11	
*Relationship to child with 22q11DS*		
Biological mother	80	88.9
Biological father	2	2.2
Adoptive mother	5	5.6
Legal guardian	2	2.2
Aunt	1	1.1
*Marital status*		
Married	67	74.4
Never married	7	7.8
Domestic partnership	6	6.7
Divorced	8	8.9
Separated	2	2.2
*Education*		
High school	15	16.7
Occupational/Technical/Vocational training	16	17.8
University degree (Bachelor or higher)	54	60
Other	5	5.6
*Employment*		
Full-time	53	58.9
Part-time	12	13.3
Stay-at-home caregiver (unpaid)	22	24.4
Other	3	3.3
*Hours spent caregiving per-week*		
0–40	21	23.3
41–80	14	15.6
81–120	15	16.7
121–160	15	16.7
> 161	25	27.8

22q11DS = 22q11 Deletion Syndrome, *M* Mean, *SD* Standard deviation

#### Predictor

The Patient Health Questionnaire (PHQ-9) measured parent depression levels. This questionnaire is a multipurpose instrument for screening, diagnosing, monitoring and measuring the severity of depression and has been well-validated in various populations. It has good test–retest reliability (*r* = 0.737) on the total score and convergent validity (*r* = 0.610) with the Hamilton Depression Rating Scale (HAM-D) measure of depression [37].

#### Mediator

We used the Multidimensional Scale of Perceived Social Support (MSPSS) to measure caregivers’ perceived social support. The MSPSS is a 12-question standardized, valid, and widely used instrument designed to measure an individual’s perception of support from three sources: family, friends, and a significant other. It has been shown to have good internal/test–retest reliability and high validity [38].

#### Outcome

The Strength and Difficulties Questionnaire (SDQ) Parent Version assessed internalizing and externalizing symptoms in children with 22q11DS. The SDQ is a widely used emotional and behavioural screening questionnaire of 25 items completed by their proxy parents for children and youth. Twenty questions are related to difficulties, and five are related to strengths. The difficulties items assess four subscales: emotional problems, conduct problems, hyperactivity, and peer problems, which may be combined into internalizing (emotional and peer problems) and externalizing (hyperactivity and conduct problems) symptoms. The SDQ has shown effectiveness in measuring change attributable to parenting interventions [39] and has satisfactory internal consistency measurements (α = 0.73) [40].

#### Covariates

Covariates considered for analysis comprised sociodemographic items: age of caregiver, gender (woman, man, other), marital status (married, never married, domestic partnership, divorced, separated, widowed), employment status (full-time, part-time, looking for work/unemployed, stay at home caregiver, other), education (high school, occupational/technical/vocational training, university degree, other) country (Canada, USA, European country), number of children, hours spent caregiving per week, and age of child with 22q11DS.

### Statistical analysis

Participants were excluded from analysis if they did not meet the study inclusion criteria, their answers seemed untrue (i.e. impossible values), or if they clicked “prefer not to answer” to too many questions so that statistical analyses would be skewed. There were 37 (29.1%) possible participants excluded: 22 due to too many “prefer not to answer” or unanswered questions, and 15 due to impossible values (6 with impossible child age; 9 with impossible caretaking hours). Descriptive statistics were calculated for demographic information, levels of social support, and depression. Sums of scales (MSPSS, PHQ-9, SDQ) and subscales were calculated. The clinical cut-off score for the PHQ-9 used to determine clinically significant depressive symptoms was a score above 9 [41]. Continuous variables were reported as mean and standard deviation (SD), and categorical variables were reported in number and percentage. We tested for normality for all variables and found that the data was non-normally distributed. Kendall’s Tau correlations were conducted between relevant variables (depressive symptoms, social support, and child internalizing/externalizing symptoms). The mediation analyses were performed using a robust structural equation model (SEM) adapted for skewed data with an estimation method of maximum likelihood. SEM was conducted to examine the goodness of fit of the hypothesized model. The SEM measured the indirect effect between caregivers’ depressive symptoms and (1) Child internalizing symptoms and (2) Child externalizing symptoms through the mediation pathway of caregivers’ perceived social support. We tested which covariates were significant by using nested models. Significant covariates (caregiver’s age, education level, and the child’s age with 22q11DS) were controlled for in the SEM. The model fit was considered to be satisfactory if the root mean square error of approximation (RMSEA) < 0.08, comparative fit index (CFI) > 0.90, and Tucker-Lewis Index (TLI) > 0.90. Standardized path coefficients (β) were presented, and the mediation effect was assessed using a bootstrapping approach (n = 5000). Statistical significance was set at *p* < 0.05. Mediation models were visualized using the package “Lavaan” [42]. R statistical software R version 4.3.2 (Eye Holes) was used for all analyses.

## Results

### Background characteristics

The background characteristics of participants are described in Table 1. A total of 90 caregivers were included in the analysis (88 (97.8%) women, two (2.2%) men; mean age 49.3 (SD 9.3); 56.7% from the USA, 38.9% from Canada, and 4.4% from Europe). The mean age of the child with 22q11DS was 11.8 (SD 7.6); few (n = 13) were adults. Most caregivers were biological mothers to their child with 22q11DS (88.9%), married (74.4%), with more than half having a university degree (60.0%) and/or full-time jobs (58.9%). There were 45 (50.0%) caregivers who reported spending more than 100 h per week in caregiving activities.

### Depressive symptoms, social support, internalizing and externalizing symptoms

The mean score on the PHQ-9 in caregivers was 9.12 (see Table 2), with 46 (51.1%) individuals reporting depressive symptoms in the clinically significant range (i.e., > 9). Ten (11.1%), 37 (41.1%), and 44 (48.9%) caregivers indicated low, medium and high levels of perceived social support, respectively. As determined by their caregivers’ answers to the strengths and difficulties questionnaire, 70 (77.8%) children had clinically significant internalizing symptoms and 61 (67.8%) had clinically significant externalizing symptoms.

**Table 2:** Scores on scales administered to caregivers of children with 22q11DS

Variable	Mean (SD)	Median	Possible range	Observed range
Caregivers				
Depression (PHQ-9)	9.12 (6.11)	9.00	0–27	0–26
Social Support (MSPSS) Children with 22q11DS Externalizing symptoms (SDQ) Internalizing symptoms (SDQ)	4.79 (1.38) 9.54 (3.69) 9.92 (4.01)	4.79 10.00 10.00	0–7 0–20 0–20	1–7 2–17 2–20

*SD* Standard deviation, PHQ-9 = 9-item Patient Health Questionnaire, *MSPSS* Multidimensional Scale of Perceived Social Support, *SDQ* Strengths and Difficulties Questionnaire (rated by caregiver)

### Correlations between observed variables

The results of the Kendall’s tau correlation analysis (Table 3) showed that, as expected, caregiver depressive symptoms were significantly positively correlated with both internalizing symptoms and externalizing symptoms of their children with 22q11DS. Caregiver depressive symptoms were also significantly negatively correlated with perceived levels of social support. Additionally, the caregivers’ perceived social support level was significantly negatively correlated with the child’s internalizing and externalizing symptoms.

**Table 3:** Correlations between observed variables [with confidence intervals]

Variable	1	2	3
1. Depression			
2. Social Support	− 0.44**		
	[− 0.59, − 0.25]		
3. Internalizing symptoms	0.28**	− 0.37**	
	[0.08, 0.46]	[− 0.54, − 0.18]	
4. Externalizing symptoms	0.42**	− 0.25*	0.29**
	[0.23, 0.58]	[− 0.43, − 0.04]	[0.09, 0.47]

Values in square brackets indicate the 95% confidence interval for each correlation. The confidence interval represents a plausible range of population correlations that could have caused the sample correlation. * *p* < 0.05. ** *p* < 0.01

### Testing of hypothesized models

**Fig. 1:**
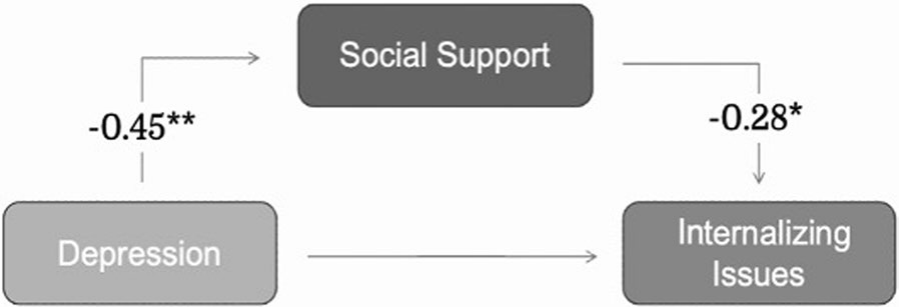
Mediation effect of caregivers’ perceived social support on caregivers’ depressive symptom rating to child internalizing symptoms. *p < 0.05, **p < 0.001. Controlled for caregiver’s age, level of education, and child age (child with 22q11DS), this analysis shows a significant mediating effect of perceived social support on the relationship between caregiver depressive symptoms and child internalizing symptoms and no significant association directly between these two parameters

**Fig. 2:**
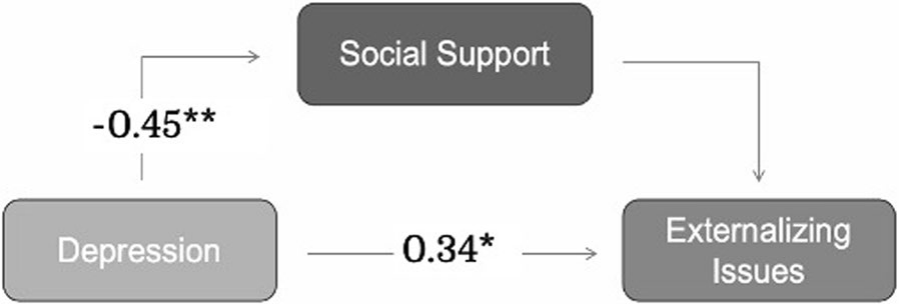
Mediation effect of caregivers’ perceived social support on caregivers’ depressive symptom rating to child externalizing symptoms. *p < 0.05, **p < 0.001. Controlled for caregiver’s age, level of education, and child age (child with 22q11DS), this analysis shows no significant mediating effect of perceived social support on the relationship between caregiver depressive symptoms and child externalizing symptoms, but there is a significant association directly between these two parameters

The hypothesized models for internalizing and externalizing symptoms (Figs. 1 and 2) were evaluated using maximum likelihood (ML) estimation.

#### Internalizing symptoms

The results of the SEM suggest that the hypothesized model for child internalizing symptoms (Fig. 1) demonstrated a good fit after controlling for significant covariates (CFI = 1, TLI = 1.156, RMSEA = 0.00, SRMR = 0.015). Significant negative associations were found between caregivers’ depressive symptoms and caregivers’ perceived social support (β = − 0.453, *p* < 0.001) and between caregivers’ perceived social support and child internalizing symptoms (β = − 0.280, *p* = 0.007). The bootstrap results indicated that caregiver depressive symptoms had an indirect effect (β = 0.127, *p* = 0.038) but no significant direct effect on child internalizing symptoms. Nearly half (41.7%) of the relationship between child internalizing symptoms and caregivers’ depression was explained by the indirect effect of caregivers’ perceived social support. This model explained 24.5% of the variance in internalizing symptoms and 25.1% of the variance in caregivers’ perceived social support.

#### Externalizing symptoms

The results of the SEM suggest that the hypothesized model for child externalizing symptoms (Fig. 2) also demonstrated a good fit after controlling for significant covariates (CFI = 1, TLI = 1.034, RMSEA = 0.00, SRMR = 0.025), explaining 26.0% of the variance in externalizing symptoms and 25.1% of the variance in caregivers’ perceived social support. The details of the results, however, differed from those for internalizing symptoms. While there was the same significant negative association between caregivers’ depressive symptoms and caregivers’ perceived social support (β = − 0.453, *p* < 0.001), there was a significant positive association between caregivers’ depressive symptoms and child externalizing symptoms (β = 0.340, *p* = 0.003). The bootstrap results indicate caregivers’ depressive symptoms had a significant direct effect (β = 0.340, *p* = 0.003) but a non-significant indirect effect (β = 0.065, *p* = 0.184) on child externalizing symptoms.

## Discussion

Using standardized measures, the current study aimed to evaluate whether caregivers’ depressive symptoms are associated with internalizing and externalizing symptoms in children with 22q11DS and whether the relationship between caregivers’ depressive symptoms and child internalizing/externalizing symptoms is mediated by their perceived levels of social support. Based on data collected from 90 participants in North America and Europe, our study revealed that caregivers’ depressive symptom rating was positively associated with externalizing symptoms in children with 22q11DS but not with internalizing symptoms once mediation via caregivers’ perceived social support was taken into account. The relationship between caregivers’ depression and internalizing symptoms of the child was fully mediated by perceived social support. This means that once the influence of perceived social support is accounted for, there is no direct effect of caregivers’ depression on the child’s internalizing symptoms—perceived social support entirely explains this relationship. In contrast, the caregivers’ perceived social support did not mediate the relationship between caregivers’ depressive symptoms and externalizing symptoms. Specifically, caregivers experiencing depressive symptoms are less likely to have reported strong social support, and this lack of perceived support seems to be associated with higher levels of internalizing symptoms in children with 22q11DS.

The prevalence of caregivers with depressive symptoms in the clinical range in our sample is relatively high (51%). Finless et al. (2024) conducted a similar study on mothers of children with 22q11DS, finding that 39% of participants experienced clinically significant depressive symptoms. The differences in findings between their study and ours may be due to sample size (71 participants versus 90 in ours) and participant inclusion criteria. Our study employed more lenient exclusion criteria and included participants who did not complete all questionnaires. In contrast, Finless et al. required responses to the Screen for Adult Anxiety-Related Disorders (SCAARED) and PTSD Checklist (PCL-5), which may have discouraged participation from individuals with depression or resulted in an incomplete survey from those experiencing more significant distress. An independent study also reported a high prevalence (26.8%) of mothers (n = 71, M = 40.5 years) of children with 22q11DS (M = 9.2 years) who screened positive for elevated levels of depression. However, comparisons are challenging given an unspecified screening tool [18]. Also, parents of children with neurodevelopmental disorders (e.g., ASD, fetal alcohol syndrome, down syndrome) are more likely to be diagnosed with depression up to decades after the birth of their child compared to parents of children without neurodevelopmental disorders [43].

The levels of caregiver-perceived social support reported here are comparable to the results of a Polish study of 44 caregivers of children with 22q11DS, reporting that 50% could always/often count on support from their family [5]. Also, we found elevated levels of both internalizing and externalizing symptoms, with internalizing symptoms higher, in line with previous literature for 22q11DS [8, 44]. However, our caregiver reported ratings of clinically significant internalizing and especially externalizing symptoms appear somewhat higher than those reported in previous studies (e.g., 48.1 and 11.1% of 12 to 18-year-olds with internalizing and externalizing symptoms, respectively, [44]), though comparisons are challenging given methodological and sample differences.

Our findings support the hypothesis that caregivers’ depressive symptoms are associated with internalizing and externalizing symptoms in children with 22q11DS, in line with studies of families with typically developing children [45] and those affected by 22q11DS [28]. However, our study further elucidates a potential pathway between parental depressive symptoms and internalizing symptoms. A novel and clinically relevant finding is that the relationship between caregiver depressive symptom ratings and internalizing symptoms in the child appears to be fully mediated by perceived social support. These findings are consistent with previous research in families with typically developing children [46].

Social support can be described as the psychological and material resources provided through social interaction [47]. There are three main types of social support: emotional support (e.g. empathetic understanding and warm care), instrumental support (concrete and tangible assistance such as services provided and practical products) and informational support (advice such as self-evaluation and problem-solving) [48]. Caregivers of children with 22q11DS could benefit from all three types of support, such as conversations with understanding peers and/or professionals (emotional support), respite/baby-sitting services (instrumental), and information about 22q11DS from healthcare.

Social support did not, however, mediate the relationship between caregivers’ depression and externalizing symptoms in their offspring with 22q11DS. This finding is consistent with a study by Taraban and colleagues that reported no effect of mothers’ social support satisfaction on the relationship between parental depressive symptoms and early childhood externalizing symptoms of their adopted offspring [49]. While we postulated that caregiver mental health would influence child symptomatology, it is also possible that the symptoms of the children with 22q11DS, especially externalizing symptoms, may be negatively influencing caregiver mental health, leading to depressive symptoms. Indeed, difficult child behaviour has been shown to be the most important predictor of a caregiver’s psychological well-being [50], with child externalizing symptoms reported to increase levels of frustration, self-criticism, and self-doubt, heightening caregiver depressive symptoms [51].

A key strength of this study is that it explored and assessed factors contributing to overall mental health in an under-researched group—families affected by 22q11DS. Caregivers of children with rare diseases are often overlooked in research. Participants were North American and European. Thus, results would be generalizable to Western cultures with access to genetic testing, health care and increased support. Future studies should assess caregiver’s depressive symptoms, perceived social support, and children’s internalizing and externalizing symptoms in families affected by 22q11DS in less developed and non-English speaking countries. Our study identified possible underlying mechanisms, specifically regarding a potentially modifiable factor, social support, in the associations between caregivers’ depressive symptom ratings and internalizing symptoms in their children with 22q11DS.

This study also has several limitations. The cross-sectional design prevented the determination of causal relationships and directionality between caregivers’ depressive symptoms, perceived social support, and their children’s internalizing and externalizing symptoms. These variables are dynamic in nature. Therefore, longitudinal studies should be conducted to validate our findings further. Use of retroactive self-report and caregiver-reported data may be affected by recall biases. However, the key variables used in the SEM were based on validated scales, which enhances the study’s potential validity. Additionally, we did not assess the severity of 22q11DS features in each child; thus, we could not assess how the severity or complexity of the disorder may have affected the relationships examined. All but two participants were mothers of children with 22q11DS. Mothers, however, remain the main caregivers for their children and tend to experience more psychological stress and depression than fathers [52, 53]. Future research should try to include more male caregivers of children with 22q11DS.

The results of this study yield several practical implications. The high prevalence of depressive symptoms among caregivers of children with 22q11DS along with high rates of child internalizing and externalizing symptoms highlight the substantial burden and stress associated with caring for a child with this complex multi-system genetic condition. Social support is known to buffer the effects of stress on mental health, and for creating positive family dynamics [24]. One way to enhance social support in families affected by 22q11DS and perhaps help mitigate internalizing symptoms in their 22q11DS offspring may be to connect families with peers with similar experiences, as favoured by families in a recent survey [36]. A tailored group and peer-supported intervention could improve the mental health of the parent and potentially also that of the affected child [36, 54–56]. Because 22q11DS is a rare disorder, caregivers of affected children often do not live near one another. An online format overcomes this challenge by enabling families worldwide to connect and support each other [36, 55]. In addition to educational and peer support, the results suggest that diagnosis and effective clinical management of treatable conditions, including those associated with externalizing symptoms, may help ameliorate caregiver burden [1, 2].

In conclusion, caregivers reporting that they experience depressive symptoms are less likely to report that they have strong social support, and this is associated with a greater likelihood of internalizing symptoms in their children with 22q11DS. In contrast, for externalizing symptoms, the association with caregiver depressive symptoms was stronger, but there was no apparent mediation by perceived social support. The results indicate the need for improved social and clinical support for families living with 22q11DS to improve the mental health of both parents/caregivers and affected children. As such, our findings may provide valuable insights for designing and implementing future interventions for families living with 22q11DS.

## Data Availability

The datasets generated during the study are available from the corresponding author upon reasonable request. The datasets used and/or analyzed for the current study are not publicly available due to privacy regulations from the institution’s Research Ethics Board.

## Abbreviations

22q11DS: 22Q11.2 deletion syndrome
ADHD: Attention-deficit hyperactivity disorder
ASD: Autism spectrum disorder
PHQ-9: Patient health questionnaire
HAM-D: Hamilton depression rating scale
MSPSS: Multidimensional scale of perceived social support
SDQ: Strengths and difficulties questionnaire
USA: United states of America
PAFAS: Parent and family adjustment scales
SEM: Structural equation model
RMSEA: Root mean square error off approximation
CFI: Comparative fit index
TLI: Tucker-Lewis index
SD: Standard deviation
ML: Maximum likelihood
SRMR: Standardized Root Mean Square Residual
SCAARED: Screen for adult anxiety-related disorders
PCL-5: PTSD checklist
